# Personal Health Information Inference Using Machine Learning on RNA Expression Data from Patients With Cancer: Algorithm Validation Study

**DOI:** 10.2196/18387

**Published:** 2020-08-10

**Authors:** Solbi Kweon, Jeong Hoon Lee, Younghee Lee, Yu Rang Park

**Affiliations:** 1 Department of Biomedical System Informatics Yonsei University College of Medicine Seoul Republic of Korea; 2 Department of Medical Engineering Asan Medical Institute of Convergence Science and Technology, Asan Medical Center University of Ulsan College of Medicine Seoul Republic of Korea; 3 Department of Biomedical Informatics University of Utah School of Medicine Salt Lake City, UT United States

**Keywords:** cancer, privacy issue, personal information, prediction, RNA sequencing, machine learning

## Abstract

**Background:**

As the need for sharing genomic data grows, privacy issues and concerns, such as the ethics surrounding data sharing and disclosure of personal information, are raised.

**Objective:**

The main purpose of this study was to verify whether genomic data is sufficient to predict a patient's personal information.

**Methods:**

RNA expression data and matched patient personal information were collected from 9538 patients in The Cancer Genome Atlas program. Five personal information variables (age, gender, race, cancer type, and cancer stage) were recorded for each patient. Four different machine learning algorithms (support vector machine, decision tree, random forest, and artificial neural network) were used to determine whether a patient's personal information could be accurately predicted from RNA expression data. Performance measurement of the prediction models was based on the accuracy and area under the receiver operating characteristic curve. We selected five cancer types (breast carcinoma, kidney renal clear cell carcinoma, head and neck squamous cell carcinoma, low-grade glioma, and lung adenocarcinoma) with large samples sizes to verify whether predictive accuracy would differ between them. We also validated the efficacy of our four machine learning models in analyzing normal samples from 593 cancer patients.

**Results:**

In most samples, personal information with high genetic relevance, such as gender and cancer type, could be predicted from RNA expression data alone. The prediction accuracies for gender and cancer type, which were the best models, were 0.93-0.99 and 0.78-0.94, respectively. Other aspects of personal information, such as age, race, and cancer stage, were difficult to predict from RNA expression data, with accuracies ranging from 0.0026-0.29, 0.76-0.96, and 0.45-0.79, respectively. Among the tested machine learning methods, the highest predictive accuracy was obtained using the support vector machine algorithm (mean accuracy 0.77), while the lowest accuracy was obtained using the random forest method (mean accuracy 0.65). Gender and race were predicted more accurately than other variables in the samples. On average, the accuracy of cancer stage prediction ranged between 0.71-0.67, while the age prediction accuracy ranged between 0.18-0.23 for the five cancer types.

**Conclusions:**

We attempted to predict patient information using RNA expression data. We found that some identifiers could be predicted, but most others could not. This study showed that personal information available from RNA expression data is limited and this information cannot be used to identify specific patients.

## Introduction

High-throughput sequencing and array technologies, such as next-generation sequencing and microarrays, can be applied to personalized genomics and for medical purposes. These technologies will enable comprehensive multiomics analysis at various levels, including genomics, transcriptomics, and proteomics. In the last decade, the ability to collect and store personal data has increased significantly. A growing number of studies around the world have used multidimensional cancer genome data sets to obtain biological insights and develop clinical applications [1]. The ability to collect and store personal data has exploded, making genomic analysis a viable method for improving diagnostic accuracy and personalized medicine.

These advances require both the collection and sharing of high-resolution genetic profiles among researchers and institutions. However, this large-scale use of detailed individual-level data raises legitimate privacy concerns. It has been proposed that genetic profiles should not be collected and shared due to the potential for privacy breaches and risk of participant identification. There are standards outlined by modern data protection laws, such as the General Data Protection Regulation in the European Union, for the anonymization of data before sharing. The new General Data Protection Regulation explores the major provisions of this new regulation with regard to processing genetic data and includes it as a special category of sensitive data [2]. The Privacy Rule of the Health Insurance Portability and Accountability Act (HIPAA) sets standards for the privacy and security of health records in the United States [3]. Public databases such as The Cancer Genome Atlas (TCGA) obtain patient consent to share their genetic data. Consent is obtained due to the possible risk of exposing patient information obtained from multiomics data [4].

We have yet to discover everything there is to learn from genomes [5]. The study of personal genome interpretation using genomic data has continued to evolve and has now reached the point of being able to explain individual characteristics [6,7]. RNA expression data, a next-generation sequencing-based method for analysis of transcription, provides valuable information on the expression of specific genes [8], and it is also considered to be sensitive data [9,10]. RNA expression analysis is performed on bulk tissue samples or cell populations. Differences in cellular RNA expression profiles are caused by various factors such as cell cycle status, differentiation, and morphologic position. Despite increasing research using genomic data, there is a lack of research to determine the appropriate level of data sharing based on the predictability of personal information.

In order to protect personal information while sharing genomic data, it is necessary to evaluate all patient information that could be revealed by genomic data only. Since 2000, numerous papers that use machine learning algorithms in genome-wide analyses have been published [11]. The aim of this study is to assess whether machine learning algorithms can use RNA expression data from a public cancer genome database (TCGA) to identify patients’ personal information.

## Methods

### Study Design

**Figure 1:**
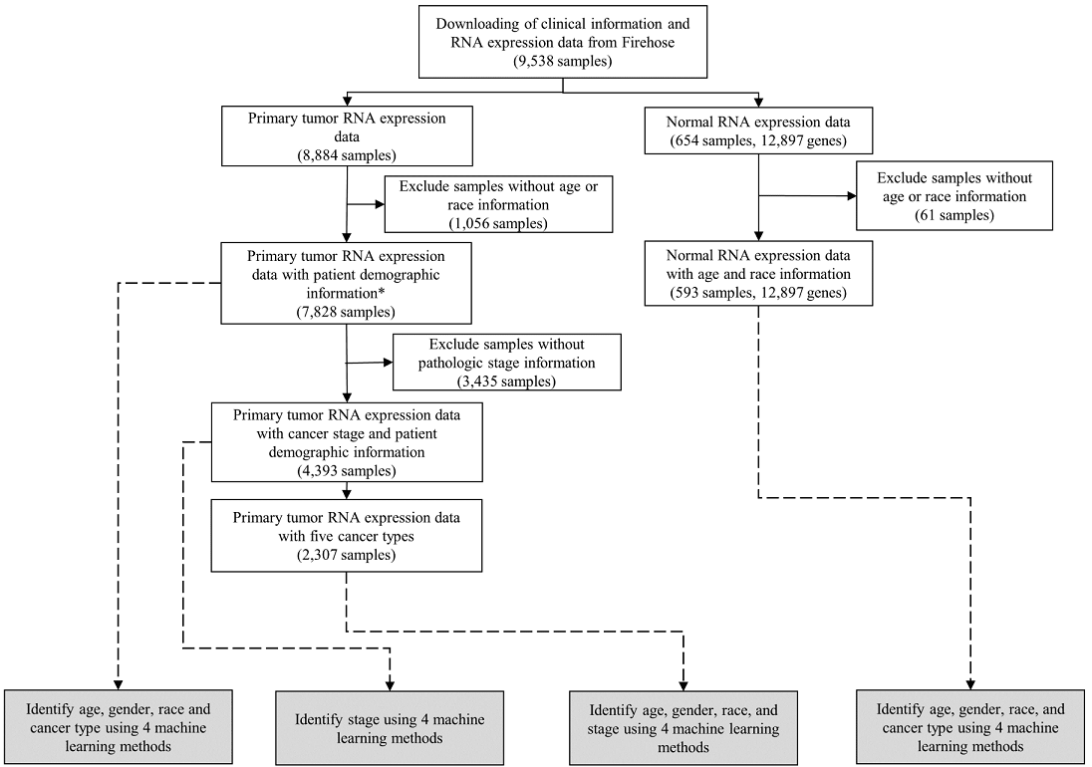
Patient inclusion and exclusion criteria (white boxes) and flowchart of the study design. The dashed lines indicate RNA expression data used to develop machine learning models. The gray boxes show machine learning models. *Demographic information: age, gender, and race.

We selected five personal data features from 9538 RNA expression samples for identification: gender, age, cancer type, race, and pathologic (cancer) stage. Information on these five features was extracted from the clinical data and separately predicted for each sample. Our data processing workflow is summarized in Figure 1. Gender consisted of two groups (male and female), age was classified into nine groups (each ranging 10 years of age), cancer type consisted of 32 groups, race consisted of five groups, and cancer stage consisted of four groups (stages I to IV). Since gender, age, cancer type, and race are typical variables, patient data with all four variables were included. However, cancer stage data were missing from many samples, and its definition differs according to cancer type, thus patient data missing this information were still included.

### Availability of Data and Materials

All RNA expression data were generated as part of The Cancer Genome Atlas [12]. These data were obtained from the FireBrowse website [13].

### Database

Gene expression information (level 3) from TCGA was downloaded from the Firehose analysis infrastructure (Broad Institute Genome Data Analysis Center). The RNA expression level 3 data contained reads per kilobase million mapped reads [14], RNA expression by expectation-maximization [15], read count, and clinical data. The TCGA level 3 RNA expression data set contained quantifications of transcript levels by normalized counts calculated using the expectation-maximization method.

We used 7828 primary tumor samples from 32 cancer types and 593 normal samples from 22 cancer types. Samples where clinical data did not exist were excluded. We used HiSeq (HiSeq 2000; Illumina Inc) RNA expression values for 12,897 genes (12,883 genes excluding the expression values of the genes on the Y chromosome) in our machine learning platforms to predict gender. In addition to the data set with samples from multiple cancer types, we created five separate data sets, each consisting of samples from a single cancer, in order to compare predictability between cancer types. These five data sets represented breast carcinoma (957/7828, 12.24%), kidney renal clear cell carcinoma (519/7828, 6.64%), head and neck squamous cell carcinoma (496/7828, 6.35%), low-grade glioma (486/7828, 6.23%), and lung adenocarcinoma (416/7828, 5.34%). These data sets were used to determine whether personal information was able to be predicted in each cancer type. Specifically, the data sets were used to determine whether the stage was predictable and how the prediction accuracy of other identifiers would change.

### Processing of RNA-Sequence Data

Level 3 RNA expression data processing and quality control were performed by the Broad Institute TCGA workgroup. Data were processed in R (version 3.6.0; packages: edgeR, limma). Expectation maximization–normalized data were preprocessed using the DGEList function (edgeR), and only genes expressed with counts per million above zero in at least 20% of samples were retained using the CPM function (edgeR) [16]. Level 3 RNA expression data were normalized within each cancer type. We performed preprocessing of level 3 RNA expression data of primary tumor samples and normal samples using the voom function (limma), which is also an alternative variance-stabilizing multiple-testing framework for RNA expression data [17]. Comparison of gene expression between cancer types was performed using linear regression models and the transformed data were then used to derive the final differential gene expression list (voom; limma). Our integrated data set contained expression values of 12,897 genes from 9538 samples.

### Personal Health Identifiers

Under the HIPAA privacy rules, personal information typically includes information that can be used to identify or track an individual, such as their name, social security number, or biometric records, either alone or in combination with other information linkable to a specific individual, such as a date or place of birth [18]. Among the available patient data, we selected five informative features that could be connected to a specific individual. HIPAA provides 18 identifiers; however, the personal information identifiers provided by TCGA are restricted. There were 29 variables associated with TCGA patient information data, from which we excluded the sample barcode, version, and survival variables. Additionally, variables missing more than 60% of data were excluded. TCGA-provided identifiers existed only for age, and we added the demographic information (ie, gender, race, stage, and cancer type) to the personal information.

### Selection of Significant Genes for Predicting Personal Information

The genes in the primary tumor data set relating to gender were analyzed using two-tailed *t* tests [19,20] with Bonferroni correction for any two-group comparisons. Other genes in the primary tumor data set relating to the remaining variables were analyzed using a one-way analysis of variance (ANOVA) [21,22] and Bonferroni posthoc tests for multiple comparisons. In addition to the 12,897 genes in the primary tumor data set, which included RNA gene expression levels, we created two data sets for each gene (*P* value≤.01) based on the *P* values of the ANOVA and *t* tests (Multimedia Appendix 1). The purpose of creating these data sets was to provide alternative data sets for evaluating whether personal information could be identified by selecting significant genes.

### Supervised Machine Learning Algorithms

We used four different supervised machine learning algorithms to generate classification models. Support vector machines are a group of related supervised learning methods used for classification and regression [23,24]. Decision tree structures use leaves to represent classifications, while branches represent conjunctions of features that lead to those classifications [23,25]. Random forest is a classifier consisting of many decision trees and determines the class, which is the mode of classes generated, by individual trees [25,26]. Artificial neural networks are an interconnected group of nodes that use a computational model for information processing. Its structure changes based on external or internal information that flows through the network. Artificial neural networks can be used to model complex relationships between inputs and outputs and find patterns in data [25,27].

The four supervised machine learning algorithms were trained on the five features subsets and cross-validated. Random forest models were generated using 100 trees. The support vector machines used linear kernels while artificial neural networks used four-layer networks. To compare the models of the four supervised machine learning algorithms, the study population was randomly stratified and split into 70% training and 30% independent testing data sets.

### Performance Evaluation

To evaluate the generated prediction models, we employed various metrics recommended for evaluating classifier performance such as accuracy, precision, recall, F1 score, and area under the receiver operating characteristic curve (AUROC). The multiclass AUROC was the mean of several AUROC classes. Quantitative measures of accuracy and AUROC were used to assess the overall performance of each classifier. AUROC is a measure of model performance, which is based on the receiver operating characteristic curve that plots the tradeoff between sensitivity and specificity of these values using commonly accepted criteria [27].

## Results

### Description of the RNA Expression Data Set

A total of 7828 primary tumor samples consisting of 32 cancer types were collected from TCGA to construct and test five personal information prediction models (Table 1). To compare tumor samples with normal samples, we extracted data from 593 normal samples from TCGA (Multimedia Appendix 2). The primary tumor data set and normal data set are structurally analogous.

**Table 1:** Personal information of the study population.

Data set information		Female, n (%)	Male, n (%)	Total, n (%)
Participants		4046 (51.69)	3782 (48.31)	7828 (100)
**Age (years)**				
	10-19	14 (0.18)	16 (0.2)	30 (0.38)
	20-29	127 (1.62)	134 (1.71)	261 (3.33)
	30-39	330 (4.22)	256 (3.27)	586 (7.49)
	40-49	629 (8.04)	451 (5.76)	1080 (13.8)
	50-59	946 (12.08)	882 (11.27)	1828 (23.35)
	60-69	995 (12.71)	1100 (14.05)	2095 (26.76)
	70-79	772 (9.86)	737 (9.41)	1509 (19.27)
	80-89	225 (2.87)	200 (2.55)	425 (5.42)
	90+	8 (0.1)	6 (0.08)	14 (0.18)
**Race**				
	American or Alaska Native	13 (0.17)	7 (0.09)	20 (0.26)
	Asian	253 (3.23)	346 (4.42)	599 (7.65)
	Black or African American	519 (6.63)	261 (3.33)	780 (9.96)
	Hawaiian or Pacific Islander	6 (0.08)	1 (0.01)	7 (0.09)
	Caucasian	3255 (41.58)	3167 (40.46)	6422 (82.04)
**Cancer stage^a^**				
	Stage I	738 (16.8)	638 (14.52)	1376 (31.32)
	Stage II	866 (19.71)	552 (12.57)	1418 (32.28)
	Stage III	569 (12.95)	491 (11.18)	1060 (24.13)
	Stage IV	184 (4.19)	355 (8.08)	539 (12.27)

^a^The percentages may not add up to 100% because of missing values.

### Prediction of the Five Personal Variables Using Four Machine Learning Algorithms in Multiple Cancers

**Figure 2:**
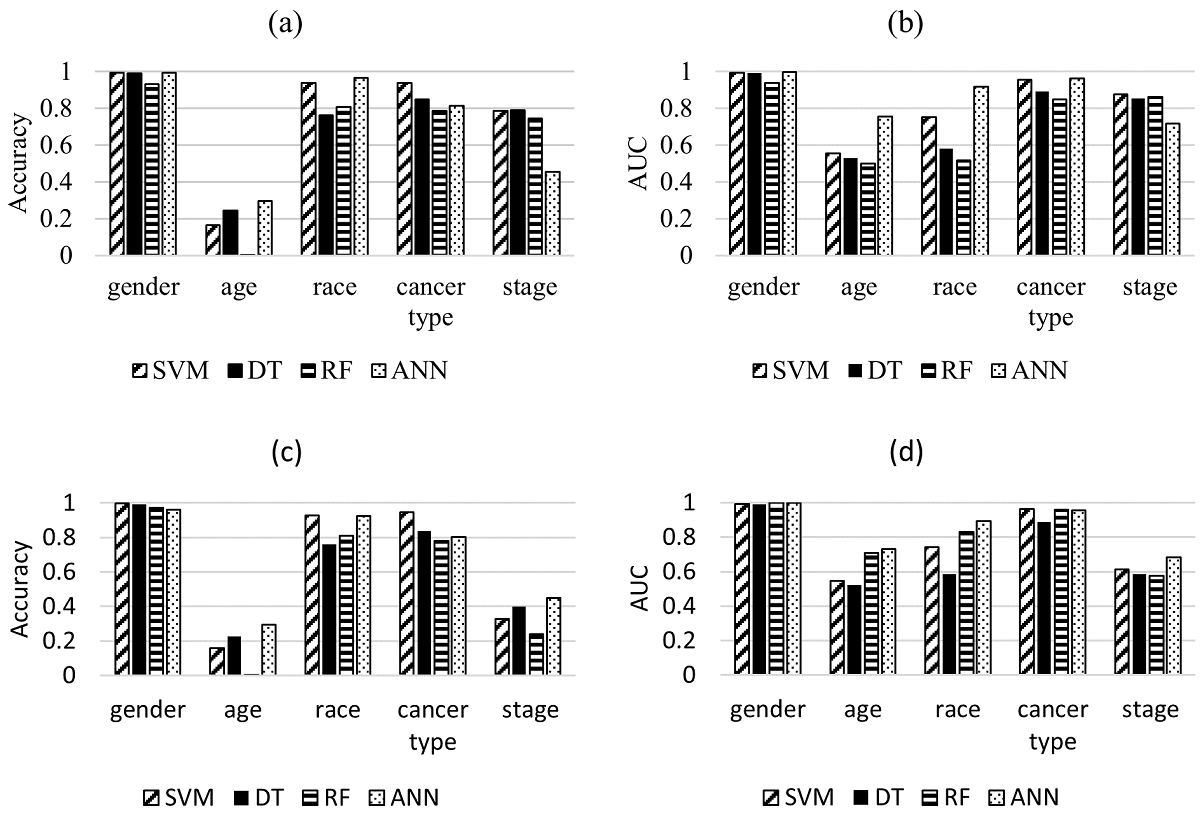
Prediction performance of personal identifiers according to independent gene sets: (a) accuracy and (b) AUROC of the personal information classifiers from a training dataset consisting of 12,897 genes; (c) accuracy and (d) AUROC of the personal information classifiers as analyzed by prediction models made from a training dataset consisting of significant genes selected through statistical analysis. ANN: artificial neural network; DT: decision tree; RF: random forest; SVM: support vector machine.

Figure 2 presents the performance of the four machine learning algorithms using 12,897 genes to predict target outcomes. The accuracies of the five personal information variables in the independent data set were 0.93-0.99 (gender), 0.0026-0.29 (age), 0.76-0.96 (race), 0.78-0.94 (cancer type), and 0.45-0.79 (stage). The AUROC of the five personal information variables in the independent data set were 0.94-0.99 (gender), 0.50-0.75 (age), 0.51-0.91 (race), 0.84-0.96 (cancer type), and 0.71-0.87 (stage). The accuracy of all models in predicting the five personal information variables ranged variedly, the lowest being in random forest and the highest being in support vector machine (Multimedia Appendix 3).

For gender, accuracy and AUROC were the highest compared to other variables: precision and recall ranges were 0.98-1.00 and 0.91-1.00, respectively. We also applied the RNA expression data of the remaining genes (excluding genes on the Y chromosome) using the machine learning algorithms, and the ranges of prediction accuracy and AUROC for gender were 0.91-0.98 and 0.96-0.99, respectively (Multimedia Appendix 4). For age, accuracy and AUROC were low, and precision and recall were less than 0.20. The accuracy and AUROC were lower for race than for gender. In the Caucasian group alone, the F1 scores were high, in the range of 0.86-0.98. However, other groups representing smaller percentages of the population were difficult to predict. Despite the multilayered structure of the 32 cancer types, the accuracy of cancer type and AUROC were lower than those of the gender variable. The accuracy and AUROC of cancer stage were lower than those of the cancer type variable, due to the multilayered structure of the 19 cancer types (Multimedia Appendix 5).

In addition to the gene data sets, results for data sets compiled with significant genes based on the *P* values were obtained. Most results were similar for all gene data sets, with accuracy and AUROC decreasing for all personal information variables, except for the cancer stage variable (Figure 2, Multimedia Appendix 5, and Multimedia Appendix 6). Thus, personal information variables such as age, cancer stage, race, cancer type, and gender were difficult to predict using the prediction models generated by machine learning.

### Prediction of Personal Information Using Four Machine Learning Algorithms in the Top Five Cancer Types

**Figure 3:**
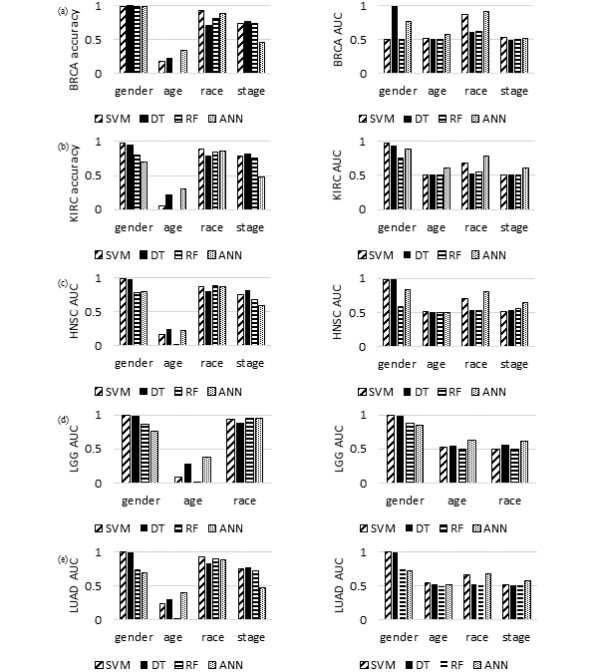
Results of model evaluation based on accuracy and AUROC for one type of cancer: (a) breast carcinoma, (b) kidney renal clear cell carcinoma, (c) head and neck squamous cell carcinoma, (d) low-grade glioma, and (e) lung adenocarcinoma. ANN: artificial neural network; DT: decision tree; RF: random forest; SVM: support vector machine.

We selected five types of cancers with large samples sizes from the primary tumor data set and developed predictive models for the four personal information variables from each cancer data set (Multimedia Appendix 7). To achieve this, we generated data sets for breast carcinoma, kidney renal clear cell carcinoma, head and neck squamous cell carcinoma, low-grade glioma, and lung adenocarcinoma, and predicted the personal information through machine learning algorithms (Figure 3). This analysis compared whether personal information was predicted differently depending on the type of cancer.

Gender accuracy was low and cancer stage accuracy was high when comparing the average accuracy of all cancers and the five specific cancers; when comparing the accuracy averages of the four machine learning algorithms, the lowest for gender was 0.85, the lowest for age was 0.14, the lowest for race was 0.83, and the lowest for cancer stage was 0.67 (Figure 3, Multimedia Appendix 8).

### Validation Through Normal Tissue Samples

**Figure 4:**
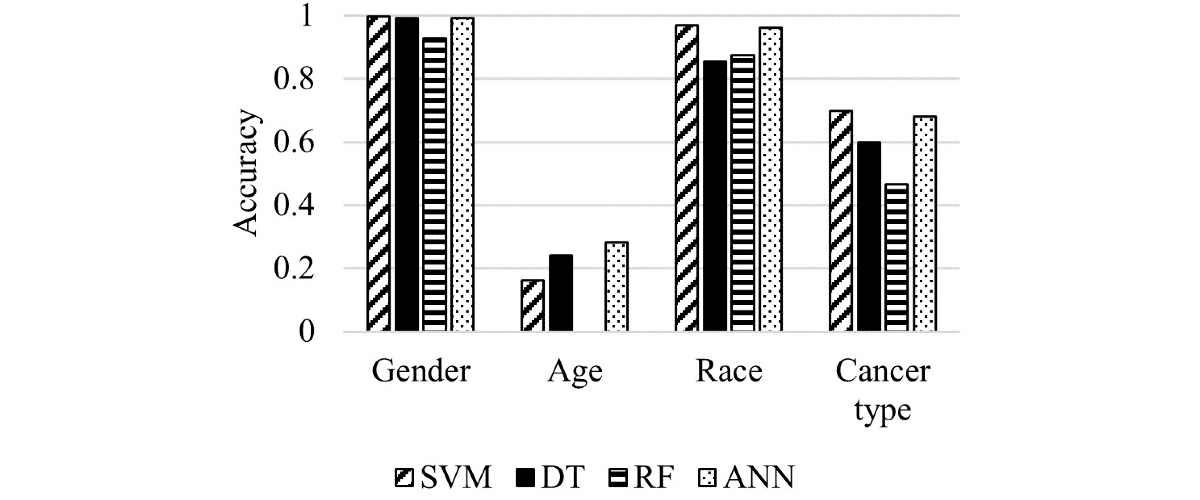
Results of model evaluation based on accuracy and AUROC when applying normal samples as a test to model personal information. ANN: artificial neural network; DT: decision tree; RF: random forest; SVM: support vector machine.

We also conducted a study to compare the predicted results for cancer samples with those for normal samples. We evaluated the optimized model using 593 normal tissue samples belonging to cancer patients from TCGA. Prediction of gender, age, and race had similar accuracies in normal samples as in tumor samples, but prediction of cancer type was less accurate in normal samples than prediction of cancer type in tumor samples (Figure 4).

### Expression of Specific Genes Depending on Personal Information

Using the RNA expression data of primary tumors, prediction models of the five personal information variables were created using four machine learning algorithms. The genes for predicting personal information were compared using important features provided by random forest and decision tree.

To facilitate the comparison of genes associated with personal information, we compared genes that play an important role when creating models for personal information retrieval. Each of the five personal information variables had a separate list of corresponding important genes; we selected the top 100 genes in these lists, based on the *P* values generated by random forest and decision tree. The results showed a low level of association between genes and personal information: only gender showed gene-relatedness in random forest. Of the top 10 genes in the list of important genes to predict the gender provided by random forest, 9 genes were located on the Y chromosome. However, of the top 10 genes in the list of important genes to predict the gender provided by decision tree, only 2 genes were located on the Y chromosome.

## Discussion

### Principal Results

The prediction of personal information using RNA expression–based approaches is a rapidly developing subfield of cancer epigenetics that has great potential to provide accurate predictive outcomes. However, there is a risk that patient information might be disclosed, thereby limiting data collection and sharing. For accurate regulation of the use of genomic data, it is necessary to examine the possibility of private information leaking. In this study, we verified personal information predictability using RNA expression profiling and presented a new direction for studies on genomic data sharing.

When predicting personal information from RNA expression data using the four machine learning algorithms, we found that most personal information could not be predicted using RNA expression data, with the exceptions being gender and cancer type. Gender could be easily predicted by analyzing the expression of sex chromosome genes, as expected [28]. Furthermore, we confirmed whether gender could be predicted by RNA expression data of the remaining genes (excluding the genes on the Y chromosome) through machine learning. Since there is a regulatory network between genes located on chromosome X or Y and chromosome 1-22, gender could still be predicted by genes located on chromosomes 1-22 [29]. In addition, several studies have shown that there are marked differences in RNA expression between different cancer types [30-32]. Personal information can be more accurately predicted when combined with other information [33], which slightly increased the accuracy of the prediction when information about cancer type was provided (increased by 0.004-0.14). However, it is still difficult to say that personal information, such as age, race, and cancer stage, could be predicted.

Aging is a very complex process that is influenced by various genetic, lifestyle, and environmental factors [34]. It causes a variety of molecular modifications and adjustments in tissues and organs that accumulate over an individual’s lifetime, including chemical modifications and alterations to gene expression [35]. Thus, it is difficult to predict age from RNA expression data alone, because of the influence of these molecular modifications and adjustments throughout the individual’s lifetime. Although HIPAA describes birth date as an identifiable variable, it is an identifier virtually impossible to predict using RNA expression data. To predict a patient's age, epigenomic data such as DNA methylation profiles, which reflects the patient's age, should be used [34,35].

The race data set was not suitable for the machine learning algorithms because the sample ratios between group populations were unbalanced. Caucasians accounted for 82% of patient samples in the RNA expression data set. For this reason, the accuracy and recall results were higher for the Caucasian group and lower for the other groups. Machine learning algorithms can suffer a performance bias in relation to classification when data sets are unbalanced [36]. On the other hand, with regard to the cancer stage data set, predictions had low accuracy across all machine learning platforms, findings that coincide with those of other studies [37,38].

Genomic data are sensitive data that can be used to identify individuals or for other purposes [39,40]. The individuality of some of the genomic data has been verified [34,35,41], but little has been studied for RNA data. Our study is thus the first to verify individual identification in RNA expression data testing the identification of personal information using RNA expression data provided by the public database TCGA. The prediction was rarely successful.

This study is the first paper to suggest determining the level of data sharing should be based on predictability of personal information. Clearly, data sharing will have a pivotal role in precision oncology. By promoting cancer genomic data sharing, researchers and clinicians will gather the information needed to improve our understanding of cancer genome and improve patient care and outcomes. Currently, projects that use genomic data use complex procedures to collect the genomic data or use previously published genomic data. Genomic data collected for a given project is often difficult to reuse in other projects. We believe that studying whether the personal information of the patient can be predicted when genomic data is shared can help determine the appropriate level of genome data sharing.

### Limitations

Our study has several limitations. First, the study was limited in terms of scope of personal information studied; we used RNA expression data and machine learning algorithms to perform predictions regarding only five personal information variables. Limited information to predict personal characteristics can be retrieved from the available TCGA data. Thus, large-scale studies employing personal information are needed to gain further insights in this field. Second, the race data were unbalanced in the TCGA primary tumor data set, with predominantly Caucasian data. Therefore, the predictive ability of the machine learning algorithms in relation to race might be reduced or biased. To clarify whether RNA expression data can predict race, further research using genomic data from diverse and balanced races is needed. Third, this study used only RNA expression data to assess the predictability of personal information using machine learning algorithms. Future research should explore the possibility of predicting personal information using other genomic information, such as DNA methylation data. Fourth, this study was done without considering clinical data, because there was little clinical information in the TCGA public database. Further research will be needed considering clinical data is sensitive information.

### Conclusions

In this study, we analyzed the ability of RNA expression data to predict patients’ personal information using machine learning algorithms. We verified that RNA expression alone is not sufficient to identify personal information using the analysis techniques employed herein. These tentative conclusions await further validation by future similar studies.

## Appendix

Multimedia Appendix 1 Three data sets with *t* test and ANOVA.

Multimedia Appendix 2 Normal samples of the four personal information in TCGA.

Multimedia Appendix 3 Receiver operating characteristics curve of support vector machine, decision tree, random forest, artificial neural network for predicting Personal Healthcare Identifiers. (a) gender, (b) age, (c) race, (d) cancer type, and (e) stage on the primary tumor test-set.

Multimedia Appendix 4 Prediction performance of gender according to an independent gene set consisting of genes located on chromosomes 1-22 and X.

Multimedia Appendix 5 Prediction performance of personal information according to independent gene sets consisting of 12,897 genes.

Multimedia Appendix 6 Prediction performances of personal information according to independent gene sets consisting of significant genes selected through statistical analysis.

Multimedia Appendix 7 Five types of cancers with large sample sizes from the primary tumor dataset.

Multimedia Appendix 8 Statistical analysis of predictive performance of personal information for five cancer types.

## Abbreviations

AUROC: area under the receiver operating characteristic curve
HIPAA: Health Insurance Portability And Accountability Act
TCGA: The Cancer Genome Atlas.
