# Supplementary material for: Personal Health Information Inference Using Machine Learning on RNA Expression Data from Patients With Cancer: Algorithm Validation Study
Source: J Med Internet Res. 2020 Aug 10;22(8):e18387. doi: 10.2196/18387 (PMC7445622; doi:10.2196/18387)
Supplement: Multimedia Appendix 1 [file jmir_v22i8e18387_app1.pdf]

Three datasets with T-test and ANOVA

Model	Feature	Sample, n	p value* (gene, n)		
			Total	<0.05	<0.01
T-test	Gender	7,828	12,897	6,186	5,798
ANOVA	Age	7,828	12,897	8,315	8,017
ANOVA	Race	7,828	12,897	6,000	5,542
ANOVA	Cancer type	7,828	12,897	8,917	8,646
ANOVA	Stage	5,485	12,897	7,251	6,880

* with Bonferroni's post hoc test
