# Supplementary figures and images for: Personal Health Information Inference Using Machine Learning on RNA Expression Data from Patients With Cancer: Algorithm Validation Study
Source: J Med Internet Res. 2020 Aug 10;22(8):e18387. doi: 10.2196/18387 (PMC7445622; doi:10.2196/18387)

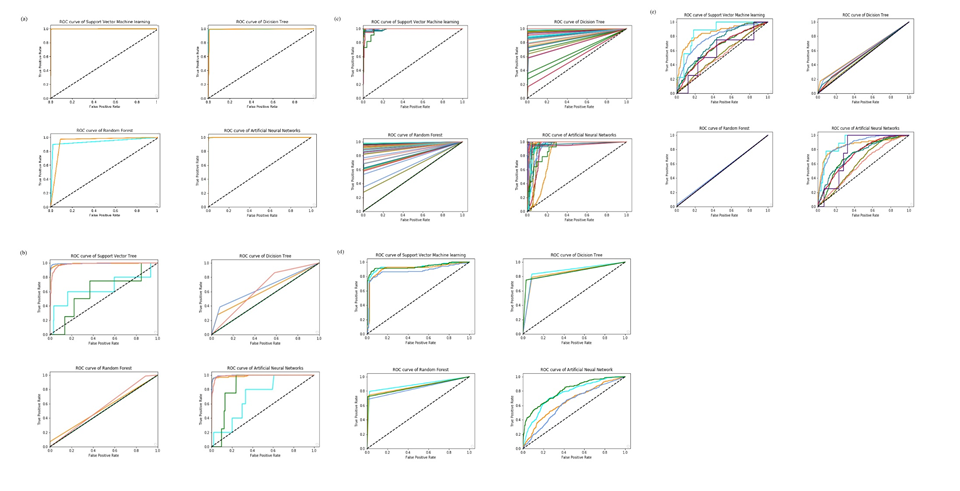

Supplement: Multimedia Appendix 3 [file jmir_v22i8e18387_app3.png]
