# Supplementary material for: Personal Health Information Inference Using Machine Learning on RNA Expression Data from Patients With Cancer: Algorithm Validation Study
Source: J Med Internet Res. 2020 Aug 10;22(8):e18387. doi: 10.2196/18387 (PMC7445622; doi:10.2196/18387)
Supplement: Multimedia Appendix 4 [file jmir_v22i8e18387_app4.pdf]

Prediction performance of gender according to an independent gene set consisting of genes located on chromosomes 1-22 and X.

Features	Genes	Machine learning algorithms								Support samples	
		Support vector machine		Decision tree		Random forest		Neural network			
		accuracy	AUC	accuracy	AUC	accuracy	AUC	accuracy	AUC	Train	Test
Gender	12,883	0.9825	0.9825	0.9634	0.9635	0.9133	0.9915	0.9662	0.9963	5,479	2,349

Features	Classes	Machine learning algorithms												Support samples	
		Support vector machine			Decision tree			Random forest			Neural network				
		precision	recall	f1-score	precision	recall	f1-score	precision	recall	f1-score	precision	recall	f1-score	Train	Test
Gender	Female	0.98	0.98	0.98	0.97	0.96	0.96	0.97	0.98	0.96	0.99	0.95	0.97	2774	1272
	Male	0.98	0.98	0.98	0.96	0.97	0.96	0.96	0.97	0.96	0.94	0.99	0.97	2705	1077

Prediction performance of gender according to an independent gene set consisting of significant genes located on chromosomes 1-22 and X selected through statistical analysis.

Features	Genes	Machine learning algorithms								Support samples	
		Support vector machine		Decision tree		Random forest		Neural network			
		accuracy	AUC	accuracy	AUC	accuracy	AUC	accuracy	AUC	Train	Test
Gender	5,790	0.9834	0.9833	0.9613	0.9613	0.9137	0.9915	0.9483	0.9950	5,479	2,349

Features	Classes	Machine learning algorithms												Support samples	
		Support vector machine			Decision tree			Random forest			Neural network				
		precision	recall	f1-score	precision	recall	f1-score	precision	recall	f1-score	precision	recall	f1-score	Train	Test
Gender	Female	0.98	0.99	0.98	0.97	0.96	0.96	0.97	0.98	0.98	0.99	0.91	0.95	2774	1272
	Male	0.99	0.98	0.98	0.96	0.96	0.96	0.98	0.97	0.97	0.91	0.99	0.95	2705	1077

Prediction performance of gender according to independent gene set consisting of genes located on chromosomes 1-22 and X by top five ranked cancer type

Cancer type	Features	Machine learning algorithms								Support samples	
		Support vector machine		Decision tree		Random forest		Neural network			
		accuracy	AUC	accuracy	AUC	accuracy	AUC	accuracy	AUC	Train	Test
BRCA	Gender	0.9861	0.5000	1.0000	1.0000	0.9861	0.5000	0.9861	0.7676	669	288
KIRC	Gender	0.9808	0.9741	0.9423	0.94	0.8077	0.7578	0.6987	0.889	363	156
HNSC	Gender	0.9866	0.9823	0.9866	0.991	0.7785	0.5897	0.7919	0.8281	347	149
LGG	Gender	1.0000	1.0000	0.9863	0.9859	0.8630	0.8756	0.7534	0.8469	340	146
LUAD	Gender	1.0000	1.0000	0.9920	0.9907	0.7280	0.7555	0.6960	0.7160	291	125

Cancer type	Features	Classes	Machine learning algorithms												Support samples	
			Support vector machine			Decision tree			Random forest			Neural network				
			precision	recall	f1-score	precision	recall	f1-score	precision	recall	f1-score	precision	recall	f1-score	Train	Test
BRCA	Gender	Female	0.98	1.00	0.99	1.00	1.00	1.00	1.00	1.00	1.00	0.97	1.00	0.99	662	284
		Male	0	0	0	1.00	0.80	0.89	1.00	0.80	0.89	0	0	0	7	4
KIRC	Gender	Female	0.96	0.90	0.93	0.89	0.90	0.89	0.91	0.95	0.93	0.90	0.40	0.55	125	58
		Male	0.94	0.98	0.96	0.94	0.93	0.93	0.97	0.94	0.95	0.68	0.97	0.80	238	98
HNSC	Gender	Female	1.00	0.88	0.93	0.98	0.94	0.96	1.00	0.96	0.98	0.50	0.03	0.06	94	38
		Male	0.94	1.00	0.97	0.97	0.99	0.98	0.98	1.00	0.99	0.68	0.99	0.81	253	111
LGG	Gender	Female	1.00	1.00	1.00	0.97	0.99	0.98	0.97	0.99	0.98	1.00	0.02	0.04	157	61
		Male	1.00	1.00	1.00	0.99	0.97	0.98	1.00	0.97	0.99	0.54	1.00	0.70	183	85
LUAD	Gender	Female	0.95	1.00	0.97	0.92	0.97	0.95	0.96	0.97	0.95	0.66	0.51	0.57	160	71
		Male	1.00	0.93	0.96	0.96	0.89	0.92	1.00	0.95	0.97	0.55	0.69	0.61	131	54
