# Supplementary material for: Personal Health Information Inference Using Machine Learning on RNA Expression Data from Patients With Cancer: Algorithm Validation Study
Source: J Med Internet Res. 2020 Aug 10;22(8):e18387. doi: 10.2196/18387 (PMC7445622; doi:10.2196/18387)
Supplement: Multimedia Appendix 5 [file jmir_v22i8e18387_app5.pdf]

Prediction performance of identifier according to independent gene set consisting of 12,897 genes.

Features	Classes	Machine learning algorithms												Support samples	
		Support vector machine			Decision tree			Random forest			Neural network				
		precision	recall	f1-score	precision	recall	f1-score	precision	recall	f1-score	precision	recall	f1-score	Train	Test
Gender	Female	1	0.99	0.99	1	Train	Test	0.98	0.91	0.94	0.99	1.00	0.99	2774	1272
	Male	0.99	1	0.99	0.99	0.99	0.99	0.9	0.97	0.94	1.00	0.99	0.99	2705	1077
Age	10	0	0.22	0.01	0	0	0	0	1	0.01	0	0	0	21	9
	20	0.32	0.25	0.28	0.17	0.17	0.17	0	0	0	0	0	0	185	76
	30	0.31	0.21	0.25	0.2	0.19	0.2	0.45	0.03	0.05	0	0	0	401	185
	40	0.27	0.26	0.26	0.18	0.21	0.19	0	0	0	0.27	0.60	0.37	773	307
	50	0.24	0.22	0.23	0.27	0.29	0.28	0	0	0	0.28	0.41	0.34	1279	549
	60	0.32	0.24	0.28	0.29	0.28	0.28	0.5	0	0	0.33	0.45	0.38	1451	644
	70	0.34	0.21	0.26	0.27	0.23	0.25	0	0	0	0	0	0	1049	460
	80	0.07	0.02	0.03	0.11	0.12	0.12	0	0	0	0	0	0	310	115
	90	0	0	0	0	0	0	0	0	0	0	0	0	10	4
Race	NATIVE AMERICAN OR ALASKA NATIVE	0.02	0.2	0.03	0	0	0	0	0	0	0	0	0	15	5
	ASIAN	0.94	0.85	0.89	0.3	0.28	0.29	0.82	0.07	0.13	0.82	0.96	0.89	405	194
	BLACK	0.97	0.84	0.9	0.37	0.39	0.38	1	0	0.01	0.99	0.87	0.92	536	244
	NATIVE HAWAIIAN	0	0	0	0	0	0	0	0	0	0	0	0	3	4
	WHITE	0.98	0.98	0.98	0.86	0.86	0.86	0.83	0.99	0.9	0.98	0.99	0.98	4520	1902
Cancer type	ACC	1	1	1	0.9	0.86	0.88	1	0.68	0.81	0	0	0	46	22
	BLCA	0.67	1	0.8	0.73	0.8	0.76	0.21	1	0.34	0.81	0.87	0.84	256	127
	BRCA	1	0.99	0.99	0.95	0.95	0.95	1	0.91	0.95	1.00	0.99	0.99	661	297

	CESC	1	0.92	0.96	0.66	0.58	0.62	1	0.35	0.51	0	0	0	163	94
	CHOL	0.5	0.45	0.48	0.33	0.27	0.3	0	0	0	0	0	0	25	11
	COAD	0.87	0.8	0.83	0.69	0.67	0.68	0.78	0.69	0.73	0.73	0.96	0.83	176	78
	DLBC	1	1	1	0.79	0.79	0.79	1	0.86	0.92	0	0	0	26	20
	ESCA	0.97	0.75	0.85	0.65	0.64	0.64	1	0.27	0.43	0	0	0	107	46
	GBM	1	0.96	0.98	0.96	0.98	0.97	1	0.92	0.96	0.89	0.15	0.26	94	46
	HNSC	0.99	0.99	0.99	0.88	0.95	0.91	1	0.93	0.97	0.92	0.99	0.95	357	140
	KICH	0.89	0.89	0.89	0.58	0.58	0.58	0.85	0.58	0.69	0	0	0	42	22
	KIRC	0.97	0.95	0.96	0.86	0.91	0.89	0.97	0.89	0.93	0.94	0.94	0.94	371	149
	KIRP	0.96	0.93	0.95	0.86	0.88	0.87	0.96	0.83	0.89	0.75	0.90	0.82	199	71
	LGG	0.99	0.98	0.99	0.99	0.98	0.98	0.98	0.98	0.98	0.75	0.99	0.85	366	122
	LIHC	0.99	0.92	0.96	0.99	0.87	0.92	1	0.87	0.93	0.94	0.98	0.96	250	95
	LUAD	0.92	0.96	0.94	0.81	0.75	0.78	0.96	0.69	0.8	0.72	0.93	0.81	284	134
	LUSC	0.97	0.84	0.9	0.7	0.73	0.71	1	0.53	0.69	0.58	0.85	0.69	258	121
	MESO	0.96	0.9	0.93	0.86	0.86	0.86	1	0.62	0.77	0.00	0.00		61	21
	OV	1	1	1	0.95	0.96	0.96	1	0.96	0.98	0.96	1.00	0.98	194	80
	PAAD	1	0.98	0.99	0.75	0.78	0.76	1	0.74	0.85	0.86	1.00	0.93	124	50
	PCPG	1	1	1	0.87	0.93	0.9	1	0.95	0.98	0.88	1.00	0.94	130	42
	PRAD	1	1	1	0.98	0.98	0.98	1	0.98	0.99	1.00	1.00	1.00	101	52
	READ	0.48	0.46	0.47	0.17	0.17	0.17	0	0	0	0	0.00	0.00	58	23
	SARC	0.96	0.99	0.97	0.93	0.89	0.91	0.98	0.9	0.94	0.78	0.99	0.87	165	83
	SKCM	1	0.88	0.94	0.58	0.73	0.64	1	0.62	0.76	0.37	0.85	0.51	68	33
	STAD	0.94	0.98	0.96	0.86	0.84	0.85	0.91	0.85	0.88	0.72	1.00	0.84	251	103
	TGCT	1	1	1	0.91	0.95	0.93	1	0.82	0.9	0.26	0.14	0.18	96	32
	THCA	1	1	1	0.99	0.98	0.99	1	0.98	0.99	1.00	1.00	1.00	284	120
	THYM	1	0.97	0.99	1	0.85	0.92	1	0.77	0.87	0.66	0.97	0.78	75	42
	UCEC	0.87	0.92	0.89	0.82	0.84	0.83	0.96	0.59	0.73	0.84	0.84	0.84	108	45

	UCS	1	0.64	0.78	0.29	0.36	0.32	0	0	0	0.67	0.14	0.24	42	14
	UVM	0.9	1	0.95	0.9	1	0.95	1	0.94	0.97	0	0	0	41	14
Stage	Stage I	0.77	0.9	0.83	0.82	0.83	0.82	0.64	0.99	0.78	0.46	0.81	0.58	960	416
	Stage II	0.84	0.81	0.82	0.82	0.79	0.8	0.96	0.74	0.84	0.54	0.28	0.37	974	444
	Stage III	0.84	0.76	0.8	0.72	0.76	0.74	0.99	0.68	0.81	0.32	0.21	0.25	775	285
	Stage IV	0.9	0.77	0.83	0.78	0.75	0.77	0.95	0.72	0.82	0.49	0.46	0.47	366	173
