# Supplementary material for: Personal Health Information Inference Using Machine Learning on RNA Expression Data from Patients With Cancer: Algorithm Validation Study
Source: J Med Internet Res. 2020 Aug 10;22(8):e18387. doi: 10.2196/18387 (PMC7445622; doi:10.2196/18387)
Supplement: Multimedia Appendix 7 [file jmir_v22i8e18387_app7.pdf]

Five types of cancers with large numbers of samples from the primary tumor dataset.

Features	BRCA (N=957)	KIRC (N=519)	HNSC (N=496)	LGG (N=486)	LUAD (N=416)
Gender, n (%)					
Female	946 (98.85)	183 (35.26)	132 (26.61)	218 (44.86)	231 (55.53)
Male	11 (1.15)	336 (64.74)	364 (73.39)	268 (55.14)	185 (44.47)
Age, years, n (%)					
10	0 (0)	0 (0)	0 (0)	4 (0.82)	0 (0)
20	8 (0.84)	2 (0.39)	6 (1.21)	71 (14.61)	0 (0)
30	60 (6.27)	15 (2.89)	11 (2.22)	151 (31.07)	0 (0)
40	197 (20.59)	86 (16.57)	60 (12.1)	105 (21.6)	27 (6.49)
50	258 (26.96)	136 (26.2)	142 (28.63)	89 (18.31)	91 (21.88)
60	244 (25.5)	149 (28.71)	170 (34.27)	51 (10.49)	138 (33.17)
70	135 (14.11)	107 (20.62)	77 (15.52)	15 (3.09)	135 (32.45)
80	52 (5.43)	24 (4.62)	30 (6.05)	0 (0)	25 (6.01)
90	3 (0.31)	0 (0)	0 (0)	0 (0)	0 (0)
Race, n (%)					
NATIVE AMERICAN OR ALASKA NATIVE	732 (76.49)	456 (87.86)	438 (88.31)	457 (94.03)	362 (87.02)
ASIAN	177 (18.5)	55 (10.6)	45 (9.07)	21 (4.32)	47 (11.3)
BLACK OR AFRICAN AMERICAN	48 (5.02)	8 (1.54)	11 (2.22)	8 (1.65)	7 (1.68)
WHITE	0 (0)	0 (0)	2 (0.4)	0 (0)	0 (0)
Cancer type, n (%)					
Stage I	172 (18.24)	263 (50.58)	25 (5.85)	0 (0)	228 (54.68)
Stage II	537 (56.95)	54 (10.38)	70 (16.39)	0 (0)	100 (23.98)
Stage III	217 (23.01)	121 (23.27)	73 (17.1)	0 (0)	68 (16.31)
Stage IV	17 (1.8)	82 (15.77)	259 (60.66)	0 (0)	21 (5.04)

The percent in parentheses of variable’s stage may not add up to 100% due to the missing value.
