# Supplementary material for: Personal Health Information Inference Using Machine Learning on RNA Expression Data from Patients With Cancer: Algorithm Validation Study
Source: J Med Internet Res. 2020 Aug 10;22(8):e18387. doi: 10.2196/18387 (PMC7445622; doi:10.2196/18387)
Supplement: Multimedia Appendix 8 [file jmir_v22i8e18387_app8.pdf]

Breast carcinoma's Machine learnings accuracy and AUC result table.

Features	Classes	Machine learning algorithms												Support samples	
		Support vector machine			Decision tree			Random forest			Neural network				
		precision	recall	f1-score	precision	recall	f1-score	precision	recall	f1-score	precision	recall	f1-score	Train	Test
Gender	Female	0.99	1	0.99	1	1	1	0.99	1	0.99	0.99	1	0.99	662	284
	Male	0	0	0	1	1	1	0	0	0	Nan	0	0	7	4
Age	20	0.01	0.5	0.02	0	0	0	0.01	1	0.01	Nan	0	0	6	2
	30	0.2	0.06	0.1	0.06	0.06	0.06	0	0	0	0.38	0.19	0.25	44	16
	40	0.39	0.4	0.39	0.29	0.28	0.29	0	0	0	Nan	0	0	144	53
	50	0.18	0.15	0.16	0.24	0.27	0.25	0	0	0	0.31	0.72	0.44	184	74
	60	0.35	0.23	0.27	0.28	0.24	0.26	0	0	0	0.38	0.53	0.44	164	80
	70	0.22	0.11	0.15	0.25	0.23	0.24	0	0	0	Nan	0	0	91	44
	80	0	0	0	0.06	0.06	0.06	0	0	0	Nan	0	0	34	18
	90	0	0	0	0	0	0	0	0	0	Nan	0	0	2	1
Race	ASIAN	0.67	0.83	0.74	0	0	0	0	0	0	0.22	0.17	0.19	36	12
	BLACK	0.98	0.84	0.9	0.48	0.6	0.53	0.9	0.36	0.51	0.97	0.66	0.79	127	50
	WHITE	0.96	0.98	0.97	0.86	0.77	0.82	0.86	0.94	0.9	0.90	0.98	0.94	506	226
Stage	Stage I	0.24	0.47	0.32	0.18	0.33	0.24	0.21	0.24	0.22	0.39	0.29	0.34	121	51
	Stage II	0.6	0.53	0.56	0.58	0.65	0.61	0.58	0.52	0.55	0.63	0.54	0.58	381	156
	Stage III	0.41	0.25	0.31	0	0	0	0.2	0.25	0.22	0.26	0.45	0.32	161	56
	Stage IV	0	0	0	0	0	0	0	0	0	nan	0	0	8	6

Kidney renal clear cell carcinoma's Machine learnings accuracy and AUC result table.

Features	Classes	Machine learning algorithms												Support samples	
		Support vector machine			Decision tree			Random forest			Neural network				
		precision	recall	f1-score	precision	recall	f1-score	precision	recall	f1-score	precision	recall	f1-score	Train	Test
Gender	Female	1	0.95	0.97	0.92	0.93	0.92	0.97	0.55	0.7	0.87	0.22	0.36	125	58
	Male	0.97	1	0.98	0.96	0.95	0.95	0.79	0.99	0.88	0.68	0.98	0.80	238	98
Age	20	0.01	0.5	0.02	0	0	0	0.01	1	0.03	Nan	0	0.00	0	2
	30	0	0	0	0	0	0	0	0	0	Nan	0	0.00	11	4
	40	0.2	0.04	0.07	0.2	0.22	0.21	0	0	0	0.33	0.04	0.08	63	23
	50	0.17	0.05	0.08	0.27	0.26	0.27	0	0	0	0.31	0.29	0.30	98	38
	60	0.5	0.06	0.11	0.34	0.32	0.33	0	0	0	0.35	0.24	0.29	99	50
	70	0.33	0.06	0.11	0.05	0.06	0.06	0	0	0	0.29	0.75	0.41	75	32
	80	0	0	0	0	0	0	0	0	0	Nan	0	0.00	17	7
Race	ASIAN	0	0	0	0	0	0	0.33	0.33	0.33	Nan	0	0.00	5	3
	BLACK	0.68	0.59	0.63	0.24	0.18	0.21	0.75	0.14	0.23	0.75	0.27	0.40	33	22
	WHITE	0.93	0.95	0.94	0.86	0.9	0.88	0.87	0.98	0.92	0.87	0.98	0.92	325	131
Stage	Stage I	0.51	0.38	0.43	0.53	1	0.69	0.67	0.03	0.05	0.58	0.84	0.68	170	80
	Stage II	0.12	0.25	0.16	0	0	0	0.14	0.7	0.24	0.10	0.1	0.10	33	20
	Stage III	0.22	0.38	0.28	0	0	0	0.2	0.34	0.25	0.33	0.03	0.06	91	29
	Stage IV	0	0	0	0	0	0	0	0	0	0.17	0.09	0.11	58	23

Head and neck squamous cell carcinoma’s Machine learnings accuracy and AUC result table.

Features	Classes	Machine learning algorithms												Support samples	
		Support vector machine			Decision tree			Random forest			Neural network				
		precision	recall	f1-score	precision	recall	f1-score	precision	recall	f1-score	precision	recall	f1-score	Train	Test
Gender	Female	0.97	0.97	0.97	0.95	1	0.97	0.8	0.21	0.33	0.68	0.34	0.46	94	38
	Male	0.99	0.99	0.99	1	0.98	0.99	0.78	0.98	0.87	0.81	0.95	0.87	253	111
Age	20	0	0	0	0	0	0	0.03	1	0.06	Nan	0	0	2	4
	30	0	0	0	0	0	0	0	0	0	Nan	0	0	9	2
	40	0.17	0.06	0.08	0.05	0.06	0.05	0	0	0	0.07	0.11	0.09	42	18
	50	0.44	0.32	0.37	0.3	0.23	0.26	0.5	0.07	0.12	0.25	0.34	0.29	98	44
	60	0.31	0.28	0.3	0.38	0.5	0.43	0.25	0.02	0.04	0.28	0.33	0.30	124	46
	70	0.2	0.04	0.07	0.15	0.16	0.16	0	0	0	0.2	0.08	0.11	52	25
	80	1	0.1	0.18	0	0	0	0	0	0	Nan	0	0	20	10
Race	NATIVE AMERICAN OR ALASKA NATIVE	0	0	0	0	0	0	0	0	0	0	0	0	1	1
	ASIAN	0	0	0	0	0	0	0	0	0	Nan	0	0	8	3
	BLACK	0.8	0.29	0.42	0.29	0.36	0.32	0.63	0.16	0.38	0.33	0.07	0.12	31	14
	WHITE	0.93	0.97	0.95	0.9	0.87	0.89	0.87	0.43	0.81	0.88	0.98	0.93	307	131
Stage	Stage I	0.17	0.56	0.26	0.12	0.44	0.2	0.13	0.22	0.17	Nan	0	0	14	9
	Stage II	0.3	0.16	0.21	0	0	0	0.22	0.26	0.24	0.33	0.16	0.21	48	19
	Stage III	0.22	0.1	0.13	0	0	0	0.17	0.14	0.15	0.33	0.29	0.31	46	21
	Stage IV	0.71	0.71	0.71	0.59	0.75	0.66	0.61	0.54	0.57	0.67	0.90	0.77	153	63

Low grade glioma's Machine learnings accuracy and AUC result table.

Features	Classes	Machine learning algorithms												Support samples	
		Support vector machine			Decision tree			Random forest			Neural network				
		precision	recall	f1-score	precision	recall	f1-score	precision	recall	f1-score	precision	recall	f1-score	Train	Test
Gender	Female	1	1	1	0.98	0.98	0.98	0.85	0.9	0.87	0.68	0.77	0.72	157	61
	Male	1	1	1	0.99	0.99	0.99	0.93	0.88	0.9	0.82	0.74	0.78	183	85
Age	10	0.01	0.25	0.02	0	0	0	0.03	1	0.05	nan	0	0	0	4
	20	0.6	0.38	0.46	0.17	0.44	0.25	0	0	0	nan	0	0	55	16
	30	0.56	0.09	0.16	0.63	0.4	0.49	1	0.02	0.04	0.49	0.73	0.58	96	55
	40	0	0	0	0.17	0.11	0.13	0	0	0	0.25	0.14	0.18	68	37
	50	0.25	0.07	0.11	0.16	0.36	0.22	0	0	0	0.25	0.79	0.38	75	14
	60	0	0	0	0.23	0.18	0.2	0	0	0	nan	0	0	34	17
	70	0	0	0	0	0	0	0	0	0	nan	0	0	12	3
Race	ASIAN	0	0	0	0	0	0	0	0	0	nan	0	0	4	4
	BLACK	0	0	0	0.17	0.25	0.2	0	0	0	nan	0	0	17	4
	WHITE	0.94	0.99	0.97	0.96	0.93	0.95	0.95	1	0.97	0.95	1	0.97	319	138

Lung adenocarcinoma’s Machine learnings accuracy and AUC result table.

Features	Classes	Machine learning algorithms												Support samples	
		Support vector machine			Decision tree			Random forest			Neural network				
		precision	recall	f1-score	precision	recall	f1-score	precision	recall	f1-score	precision	recall	f1-score	Train	Test
Gender	Female	1	1	1	0.99	1	0.99	0.77	0.82	0.79	0.77	0.662	0.71	160	71
	Male	1	1	1	1	0.98	0.99	0.74	0.69	0.71	0.63	0.7407	0.68	131	54
Age	40	0.04	0.33	0.07	0.12	0.17	0.14	0.05	1	0.09	Nan	0	0	21	6
	50	0.41	0.5	0.45	0.22	0.27	0.24	0	0	0	0.27	0.3077	0.29	65	26
	60	0.5	0.19	0.28	0.45	0.33	0.38	0	0	0	0.45	0.5962	0.51	86	52
	70	0.45	0.26	0.33	0.31	0.37	0.34	0.33	0.03	0.05	0.38	0.2857	0.33	100	35
	80	0	0	0	0	0	0	0	0	0	Nan	0	0	19	6
Race	ASIAN	0.25	0.5	0.33	0	0	0	0	0	0	Nan	0	0	5	2
	BLACK	0.8	0.33	0.47	0.17	0.17	0.17	0	0	0	0.4	0.1667	0.24	35	12
	WHITE	0.96	1	0.98	0.89	0.91	0.9	0.9	1	0.94	0.9	0.973	0.94	251	111
Stage	Stage I	0.54	0.95	0.69	0.49	1	0.66	0.47	0.34	0.4	0.51	0.8276	0.63	155	58
	Stage II	0.25	0.06	0.09	0	0	0	0.34	0.33	0.34	0.38	0.1667	0.23	60	36
	Stage III	0.22	0.11	0.14	0	0	0	0.16	0.26	0.2	0.22	0.1053	0.14	46	19
	Stage IV	0	0	0	0	0	0	0.1	0.17	0.12	nan	0	0	14	6
